# What Is Needed for a Successful Second Chance for Accused Researchers?

**DOI:** 10.18502/ijph.v49i10.4707

**Published:** 2020-10

**Authors:** Jung Hun LEE, Jeong Ho JEON, Sang Hee LEE

**Affiliations:** National Leading Research Laboratory, Department of Biological Sciences, Myongji University, Yongin, Gyeonggido 17058, Republic of Korea

## Dear Editor-in-Chief

May researchers were accused of (i) careless mistakes; (ii) failing to provide adequate oversight; (iii) not complying with policies on the treatment of human research participants, animal welfare or the declaration of conflicts of interests; or (iv) scientific misconduct (1). Data manipulation is never acceptable, but more than 570 researchers were accused of scientific misconduct (https://en.wikipedia.org/wiki/List_of_scientific_misconduct_incidents), and according to the survey data (2), an average of 2% of researchers admitted to fabricating, falsifying, or modifying data at least once. According to a report on Retraction Watch (see https://retractionwatch.com/2016/08/29/why-do-scientists-commit-misconduct/), some researchers accused of scientific misconduct have a history of mental health problems because they are under pressure from a competitive research environment and the Narcissistic Personality Disorder, described in the Diagnostic and Statistical Manual of Mental Disorders (DSM-5) and the alternative DSM-5 model (3), could account for many characteristics of highly competitive researchers. If accused researchers without mental health problems deserve a second chance, what is necessary for the successful chance?

First, a few university’s (or institute’s) leaders came to the rescue of accused researchers such as an Italian behavioral neuroscientist (4), although most leaders might disown accused researchers out of fear for the establishment’s reputation as seen in case of a world-renowned and accused neuroscientist at one of Germany’s leading neuroscience institutes (5). If a university’s (or institute’s) leaders would meticulously investigate all of the accused researchers’ scholarly conduct and ethical behavior in the publication of professional scientific research prior to the dismissal of accused researchers, the number of wrongly accused researchers would be reduced.

Second, even though the investigation of accused researchers revealed that they have engaged in scientific misconduct, it is necessary for us to give them the opportunity to redeem themselves, after having imposed suitable penalties and if there are prominent mentor(s) and programs to retrain errant researchers as seen in case of Yoshinori Watanabe mentored by Nobel prizewinner Paul Nurse (1). The reason is that many errant researchers’ careers span over two (or three) decades in particular scientific field(s) and have a series of impressive scientific achievements that were confirmed by their mentor(s). It is rare to give a second chance for accused (or errant) researchers, but more mentors and retraining programs are needed to avoid losing prominent researchers.

Finally, after retraining, accused researchers are likely to struggle to regain their careers, because no institution wants to give the appearance of scientific misconduct. However, as seen in case of Hwang Woo-Suk’s fake stem-cell lines (6), there is some hope they might be able to return to science. A nonprofit institute, Sooam Biotech Research Foundation (Seoul, Korea), helped him return to research in advanced biotechnology using animal cloning and pluripotent stem cells combined with transgenic technology. If more university’s (or institute’s) leaders were willing to help the accused and then retrained researchers continue their researches, more accused and retrained researchers would keep regaining their careers. The reason is that scientific mistake should not be done, but allowing researchers to rectify mistakes must be part of a scientific culture.

**Fig. 1:**
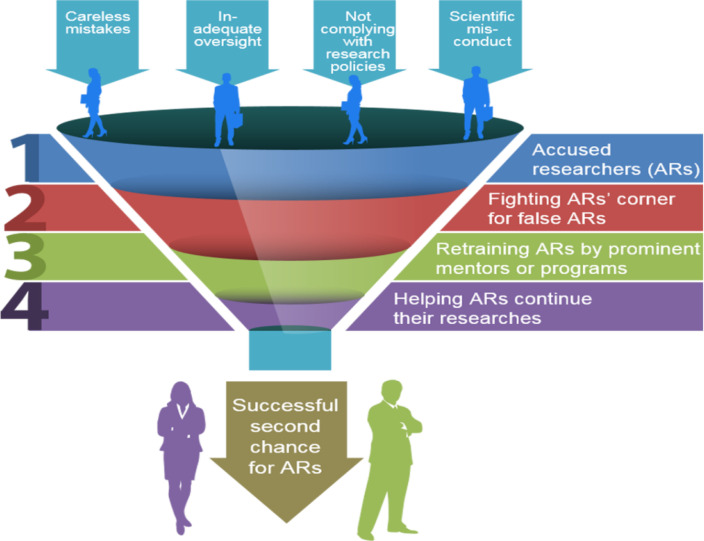
Action plan for the successful chance for accused (or errant) researchers, if accused researchers without mental health problems deserve a second chance

In summary, the following action plan (Fig. 1) is urgently needed for the successful chance for accused (or errant) researchers, if accused researchers deserve a second chance: (i) university’s (or institute’s) leaders have to fight a researcher’s corner for wrongly accused researcher; (ii) more mentors and retraining programs are needed to avoid losing prominent and errant researchers after having imposed suitable penalties; and then (iii) more university’s (or institute’s) leaders have to help the accused and then retrained researchers continue their researches.
